# Occupational biomechanical risk factors for carpal tunnel syndrome surgery: a prospective cohort study on 203 866 Swedish male construction workers followed for 19 years

**DOI:** 10.1136/oemed-2024-110008

**Published:** 2025-08-07

**Authors:** Albin Stjernbrandt, Per Liv, Jennie A Jackson, Hans Pettersson, Charlotte Lewis, Laura Punnett, Jens Wahlström

**Affiliations:** 1 Department of Epidemiology and Global Health, Umeå University, Umeå, Sweden; 2 Department of Public Health and Clinical Medicine, Umeå University, Umeå, Sweden; 3 Department of Occupational Health, Psychology and Sports Sciences, University of Gävle, Gävle, Sweden; 4 Department of Biomedical Engineering, University of Massachusetts Lowell, Lowell, Massachusetts, USA

**Keywords:** Occupational Health, Vibration, Ergonomics

## Abstract

**Objectives:**

To prospectively determine the association between occupational biomechanical exposures and the incidence of surgically treated carpal tunnel syndrome (CTS) in Swedish male construction workers.

**Methods:**

A cohort of 203 866 Swedish male construction workers who participated in a national occupational health surveillance programme between 1971 and 1993 were followed for CTS surgery between 2001 and 2019. Age, height, weight, smoking status and construction trade were obtained from programme records. CTS surgery cases were defined using the diagnostic code for CTS and surgical procedure code for peripheral median nerve decompression in the Swedish National Patient Register. Biomechanical exposure estimates were assigned by trade from a job-exposure matrix. The relative risk (RR) of CTS surgery for each biomechanical exposure was assessed with multivariable negative binomial regression modelling.

**Results:**

The study included 3851 cases and the total incidence rate of CTS surgery was 137.6 cases per 100 000 person-years. Associations were found for upper extremity load (RR 2.6; 95% CI 2.2 to 3.0), repetitive wrist flexion and extension (RR 2.6; 95% CI 2.2 to 3.0), full wrist extension (RR 2.3; 95% CI 1.9 to 2.6), power grip (RR 2.5; 95% CI 2.2 to 2.9), pinch grip (RR 2.0; 95% CI 1.7 to 2.4), handheld tool use (RR 2.3; 95% CI 2.0 to 2.7) and hand-arm vibration exposure (RR 2.3; 95% CI 1.9 to 2.7).

**Conclusions:**

Occupational upper extremity load and postural exposures were associated with increased risk for surgical treatment for CTS in this large construction worker cohort. Preventive actions and consideration of occupation on assessment are warranted.

**WHAT IS ALREADY KNOWN ON THIS TOPIC**

- Occupational biomechanical factors play a role in the development of carpal tunnel syndrome. However, there is still a lack of prospective studies and insufficient evidence regarding the potential effects of pinch grip use and hand-arm vibration exposure.

**WHAT THIS STUDY ADDS**

- This study shows that both power and pinch grip increase the risk of carpal tunnel syndrome, but that the effects of hand-arm vibration are rather minor in comparison with other biomechanical risk factors.

**HOW THIS STUDY MIGHT AFFECT RESEARCH, PRACTICE OR POLICY**

- The results underline the need for further preventive measures among manual workers to reduce the incidence of carpal tunnel syndrome and consideration of occupation on examination.

## Introduction

 Carpal tunnel syndrome (CTS) is the most common peripheral nerve entrapment disorder globally and occurs when the median nerve is compressed at the wrist when passing below the transverse carpal ligament.^1^ While surgery is effective in relieving symptoms in many patients, it is also associated with a long period of postoperative sick leave,^2^ and up to half of surgically treated patients with CTS still require long-term modified duties.^3^ Personal risk factors for CTS include female sex, pregnancy, obesity, diabetes mellitus and other endocrine disorders, smoking, rheumatic disease or other joint disorders, previous wrist fracture or hand trauma, anthropometric measures and genetic susceptibility.^14^,^7^ The prevalence rate of CTS in the general population varies depending on diagnostic criteria, but generally ranges from about 7% to 16%^8^ with a female predominance.^7^ However, among men, CTS appears to be more often related to occupational factors, as exemplified by a study that calculated a population-attributable factor of 50% among male blue-collar workers.^9^ This implies that the burden of CTS among men could be drastically decreased with appropriate preventive measures in the workplace.

According to the literature, occupational risk factors include repetitive wrist movements, forceful gripping, hand-arm vibration (HAV), cold exposure and psychosocial factors.^10^,^15^ Previous studies have used many different definitions for forceful gripping, such as tightening with force,^16^ sustained forceful arm movements^17^ or force exertion over 1 kg.^18^ However, a recent review concluded that there are very few studies that have investigated the potential effects of pulp pinch grip (ie, opposition of the thumb towards the index and long finger) in relation to CTS.^19^ Similarly, the evidence for HAV as an independent risk factor for CTS was also considered insufficient.^19^ Many previous studies on occupational risk factors have had small sample sizes: one systematic review concluded that most previous studies included less than 100 cases, which may have led to inadequate precision in risk estimates.^15^ This notion motivates further studies with larger sample sizes. Finally, to better understand the exposure-effect latency response, there is a need for prospective studies of CTS incidence in an occupational setting.

The primary aim of our study was therefore to prospectively determine the association between occupational biomechanical exposures and the incidence of surgically treated CTS in Swedish male construction workers over a 19-year follow-up period. Secondary aims included investigating risks related to specific occupational groups as well as personal risk factors for subsequent CTS surgery.

## Methods

### Study design and setting

We conducted a prospective cohort study where the study sample was selected from a total of 389 132 Swedish construction workers who had participated in health examinations as part of a national health surveillance programme (‘Bygghälsan’) between 1971 and 1993. While participation was voluntary, at least 80% of eligible Swedish construction workers completed at least one health examination during this period and were therefore included in the database.^20^ The year of first and last attendance to the programme, as well as the number of health examinations, varied between subjects. Follow-up of participants was conducted using the outpatient surgical records of the Swedish National Patient Register between the years 2001 (when the digital register was launched) and 2019.

### Participants

**Figure 1:**
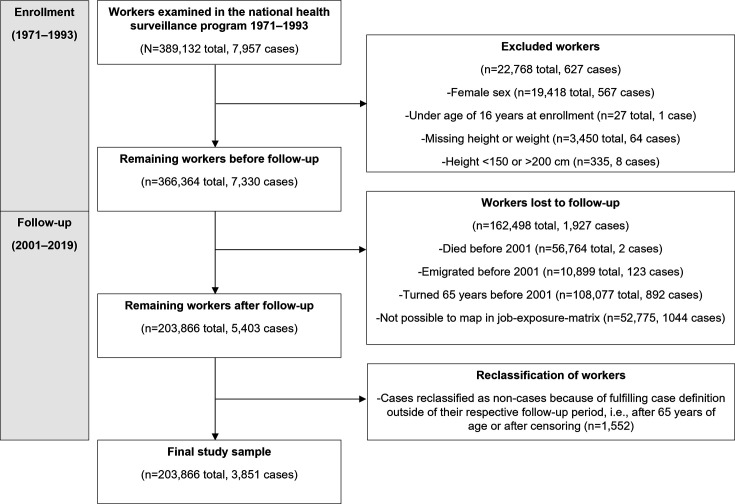
Flow chart showing the exclusion criteria and sources of data loss. A single study participant could fulfil more than one criterion within each box.

We limited our study sample to men since women only constituted about 5% of the participants in the health surveillance programme and generally lacked data on occupational title. At enrolment, we excluded workers who were younger than 16 years, unusually short (<150 cm) or tall (>200 cm) or had missing height or weight information. At follow-up, we censored for reaching retirement age (65 years), death or emigration. Data on deaths and emigration were retrieved from the Swedish Total Population Register, held by Statistics Sweden. Workers who lacked job titles were also removed since their workplace exposures could not be determined. The remaining 203 866 workers comprised the study sample. A flowchart showing the exclusion criteria and sources of data loss is presented in figure 1.

### Variables

Data on age, height, weight, smoking status and job title were retrieved from the last available health surveillance records. A subset of the participants (n=60 164) in the national health surveillance programme also responded to a written survey that was administered between 1989 and 1993. For these participants, we used data from one survey item that asked participants to rate problems (pain, aches, discomfort) in the wrist or hand during the last 12 months on a 5-point scale: never, seldom, sometimes, often or very often.

A total of 212 job titles were mapped to 21 occupational groups by occupational health service experts, and the procedure has previously been reported in detail.^21^ Based on the occupational groups, biomechanical exposure levels were determined using a job-exposure matrix (JEM) developed specifically for this cohort (online supplemental table 1). Two occupational ergonomists and one occupational physician reviewed the original ergonomic assessments that were conducted while the health surveillance programme was active and independently assigned average daily exposure intensity or frequency for the selected variables in the JEM to three categories: low, moderate or high. An occupational hygienist assessed exposure to HAV in another set of categories: none, low or high. Exposure estimates were then assigned to each study participant based on the JEM ratings for the occupational group corresponding to the job title reported at the last health examination.

CTS surgery cases were defined using the International Statistical Classification of Diseases and Related Health Problems (ICD-10) code for CTS (G56.0) together with the surgical procedure code for peripheral median nerve decompression (ACC051) from the Swedish version of the Nordic Medico-Statistical Committee Classification of Surgical Procedures (V.1.9). A single person could only be included as a case once, even if subjected to ipsilateral reoperation or having bilateral surgery at different time points.

### Statistical analysis

Incidence rates (IR) of CTS surgery were calculated for the entire observation period (2001–2019). Person-years were calculated from 2001 until surgery or the end of the observation period. For comparison purposes, we extracted the national average annual CTS surgery IR using the same surgical procedure code (ACC051) for males between 25 and 64 years from outpatient surgical records between 2005 and 2019 in the Swedish National Patient Register. The follow-up time of each worker was split into 1-year time intervals to account for effects of both age and calendar time. Negative binomial regression models with a log-link function were used to estimate relative risks (RRs) with 95% CIs for the occupational groups and biomechanical exposure categories, using unexposed foremen and white-collar workers as the reference group. All analyses on occupational groups, biomechanical exposures and survey responses were adjusted for age, height, body mass index (BMI), smoking status and calendar year. Continuous variables were adjusted using natural cubic splines with three knots, placed at the 10th, 50th and 90th percentile of the covariate. CTS surgery IR was also modelled in relation to age and height using natural cubic splines.

## Results

### Study sample

There were 203 866 male construction workers included in our final study sample (figure 1), with a mean (SD) age at first examination of 27.3 (7.1) years, height 178.7 (6.3) cm, weight 75.3 (10.6) kg and BMI 23.6 (3.0) kg/m². The mean (SD) age at the start of follow-up (2001) was 48.0 (9.8) years, ranging from 26 to 64 years. Ever smoking was reported by 100 354 (49.2%) workers. Subjects in the cohort had participated in the health examinations three times on average (range 1–13 times). A total of 3851 CTS surgery cases were identified during the follow-up period (2001–2019), with a mean (SD) age at first examination of 25.1 (6.0) years, age at CTS surgery of 53.7 (7.9) years, height 178.1 (6.4) cm, weight 77.0 (11.4) kg and BMI 24.3 (3.1) kg/m². Ever smoking was reported by 1823 (47.3%) of cases. The overall IR for CTS surgery in the study sample was 137.6 cases per 100 000 person-years (95% CI 133.3 to 142.0). The IR increased during the observation period (table 1) and with age (online supplemental figure 1). In comparison, the national male average annual IR in the Swedish National Patient Register was 88.8 per 100 000 person-years.

**Table 1:** The relative risk of carpal tunnel syndrome surgery in relation to personal factors and period

Variable*	Categories	Total number of workers	Person-years	Number of cases	Incidence rate†	Relative risk‡	95% CI
Body mass index	Underweight	3523	57 295	40	69.8	0.6	0.4 to 0.8
	Normal weight	145 420	2 073 479	2471	119.2	Reference	–
	Overweight	48 707	593 820	1157	194.8	1.6	1.5 to 1.8
	Obese	6216	73 830	183	247.9	2.1	1.8 to 2.4
Smoking status	Never	93 497	1 440 825	1889	131.1	Reference	–
	Ever	100 354	1 235 720	1823	147.5	1.1	1.1 to 1.2
	Unknown	10 015	121 878	139	114.1	0.9	0.7 to 1.03
Height (cm)	150–160	389	4682	11	235.0	1.6	0.9 to 2.9
	161–170	19 166	238 395	417	175.0	1.2	1.1 to 1.3
	171–180	105 912	1 414 961	2106	148.8	Reference	–
	181–190	71 613	1 033 956	1214	117.4	0.8	0.7 to 0.9
	191–200	6786	106 429	103	96.8	0.7	0.5 to 0.8
Period	2001–2007	203 866	1 313 025	1394	106.2	Reference	–
	2008–2013	164 601	872 446	1380	158.2	1.4	1.3 to 1.5
	2014–2019	119 072	612 534	1077	175.8	1.5	1.4 to 1.6

* Determined by the most recent recorded data from the national occupational health surveillance programme.

† Per 100 000 person-years.

‡ The relative risks for body mass index, smoking status and height are unadjusted. The periods are adjusted for age and the total number of workers exceeds the study sample size since each person could be represented in several periods.

### Personal factors

**Figure 2:**
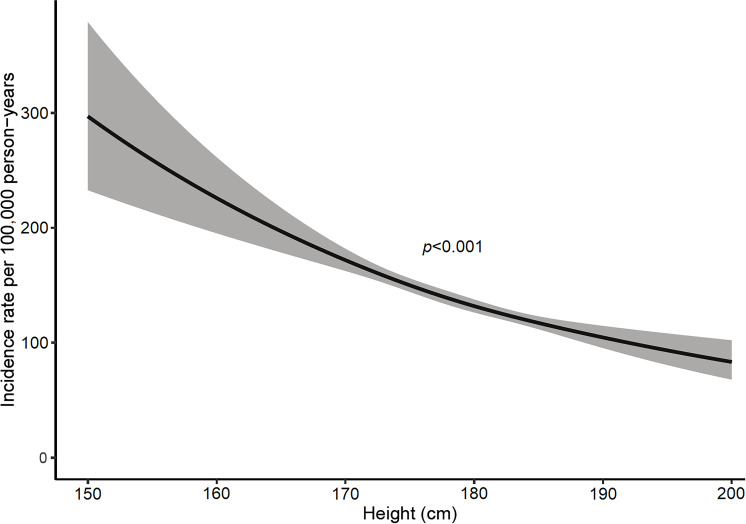
The incidence rate of surgery for carpal tunnel syndrome by height, modelled using natural cubic splines with three nodes. The association remained after adjusting for occupational group and smoking status.

The RR was elevated for overweight and obese subjects compared with normal-weight subjects and markedly below 1.0 for underweight subjects (table 1). Height showed an inverse relation, where the RR decreased monotonically from shorter to taller subjects (table 1; figure 2). There was a small increase in risk with having ever been a smoker.

### Occupational group and biomechanical exposures

Among occupational groups, most manual construction workers had an increased RR compared with foremen and white-collar workers (table 2). Only refrigerator technicians and drivers had RRs with CIs that included 1.0.

**Table 2:** The relative risk of carpal tunnel syndrome surgery by occupational group, sorted in descending order of relative risk

Occupational group*	Total number of workers	Person-years	Number of cases	Incidence rate†	Relative risk‡	95% CI
Foremen and white-collar workers	24 526	278 232	178	64.0	Reference	–
Woodworkers	47 416	691 067	1183	171.2	2.7	2.3 to 3.2
Floor layers	4122	59 080	103	174.3	2.7	2.1 to 3.5
Sheet metal workers	9457	139 069	233	167.5	2.6	2.2 to 3.2
Roofers	1008	13 284	23	173.1	2.6	1.7 to 3.9
Bricklayers	5839	76 619	124	161.8	2.5	2.0 to 3.2
Asphalt workers	2751	34 919	63	180.4	2.5	1.9 to 3.3
Concrete workers	17 905	232 284	364	156.7	2.3	1.9 to 2.8
Painters	16 856	241 093	328	136.1	2.2	1.8 to 2.7
Repairers	1782	22 627	35	154.7	2.2	1.5 to 3.2
Plumbers	16 988	233 195	329	141.1	2.2	1.8 to 2.6
Rock workers	1527	16 361	25	152.8	2.1	1.4 to 3.2
Crane operators	1972	18 818	29	154.1	2.1	1.4 to 3.1
Glass workers	2096	29 796	40	134.3	2.1	1.5 to 2.9
Preparatory workers	7178	98 333	138	140.3	2.0	1.6 to 2.5
Heavy machinery operators	7394	88 095	121	137.4	1.8	1.4 to 2.3
Insulators	2033	29 306	34	116.0	1.8	1.3 to 2.6
Electricians	29 335	449 042	453	100.9	1.6	1.4 to 2.0
Refrigerator technicians	1044	15 595	16	102.6	1.6	0.95 to 2.6
Drivers	2637	31 610	32	101.2	1.4	0.94 to 2.0

* Determined from the most recent recorded data from the national occupational health surveillance programme.

† Per 100 000 person-years.

‡ Adjusted for age, height, body mass index, smoking status and time of surgery (first or second half of the observation period).

Among occupational biomechanical exposures, increased RRs that followed positive exposure-response trends were found for intensity of upper extremity load, intensity of power grip and frequency of handheld tool use in relation to CTS surgery (table 3). The highest RR was seen in high intensity of upper extremity load (RR 2.6; 95% CI 2.2 to 3.0). Increased RRs were also found for frequency of repetitive wrist flexion and extension, frequency of full wrist extension, frequency of pinch grip use and magnitude of HAV exposure, but these did not show positive exposure-response trends. Finally, among the workers for whom self-reported symptoms were collected by survey (n=60 164), there was an increased risk of future CTS surgery for those who had experienced problems in their wrist or hand during the last 12 months both seldom/sometimes (RR 1.5; 95% CI 1.3 to 1.7) and often/very often (RR 2.1; 95% CI 1.8 to 2.5).

**Table 3:** The relative risk of carpal tunnel syndrome surgery in relation to occupational exposure variables, as determined by the job-exposure matrix

Variable	Categories	Total number of workers	Person- years	Number of cases	Incidence rate*	Relative risk†	95% CI
Foremen and white-collar workers	–	24 526	278 232	178	64.0	Reference	–
Intensity of upper extremity load	Low	12 003	138 523	182	131.4	1.7	1.4 to 2.2
	Moderate	88 936	1 273 091	1646	129.3	2.0	1.7 to 2.4
	High	78 401	1 108 577	1845	166.4	2.6	2.2 to 3.0
Frequency of repetitive wrist flexion and extension	Low	60 934	810 358	1227	151.4	2.2	1.9 to 2.6
	Moderate	46 323	682 237	782	114.6	1.8	1.5 to 2.1
	High	72 083	1 027 597	1664	161.9	2.6	2.2 to 3.0
Frequency of full wrist extension	Low	84 747	1 164 941	1731	148.6	2.3	1.9 to 2.6
	Moderate	94 593	1 355 251	1942	143.3	2.3	1.9 to 2.6
	High	0	0	0	0	–	–
Intensity of power grip	Low	14 754	173 441	245	141.3	1.9	1.6 to 2.3
	Moderate	69 197	1 004 978	1254	124.8	2.0	1.7 to 2.3
	High	95 389	1 341 772	2174	162.0	2.5	2.2 to 2.9
Frequency of pinch grip use	Low	17 386	215 885	291	134.8	1.9	1.5 to 2.2
	Moderate	98 371	1 368 687	2204	161.0	2.5	2.1 to 2.9
	High	63 583	935 619	1178	125.9	2.0	1.7 to 2.4
Frequency of handheld tool use	Low	16 850	203 237	285	140.2	1.9	1.6 to 2.3
	Moderate	16 159	224 940	326	144.9	2.2	1.8 to 2.6
	High	146 331	2 092 014	3062	146.4	2.3	2.0 to 2.7
Magnitude of hand-arm vibration exposure	None	64 719	865 761	1227	141.7	2.1	1.8 to 2.5
	Low	93 407	1 383 158	2022	146.2	2.3	2.0 to 2.7
	High	21 214	271 273	424	156.3	2.3	1.9 to 2.7

* Per 100 000 person-years.

† Adjusted for age, height, body mass index, smoking status and time of surgery (first or second half of the observation period).

## Discussion

### Main findings

This study showed that Swedish male construction workers had an elevated risk of CTS requiring surgical release compared with the general population, and increased risk was found in 17 of the 19 construction trades compared with unexposed subjects. Exposure to upper extremity load, repetitive wrist movements, extended wrists, power and pinch grip, handheld tool use and HAV increased RRs of future CTS surgery. Among personal factors, there were increases in RRs for overweight and obese individuals, ever-smokers and those of shorter stature. Finally, self-reported wrist or hand problems in the preceding years were associated with a roughly doubled RR of CTS surgery during follow-up.

### Interpretation

The IR of CTS surgery among men between 26 and 64 years at follow-up in our cohort was more than 50% higher than the official Swedish statistics for the general male population between 25 and 64 years (137.6 vs 88.8 cases per 100 000 person-years). It was also higher than the rate of CTS surgery in the male Danish general population between 15 and 64 years (55 cases per 100 000 person-years)^22^ and a male Italian population between 25 and 59 years (46.4 cases per 100 000 person-years).^23^ Although differences in age distribution limit comparisons, the results suggest that occupational factors increase the risk among the Swedish construction workers.

The JEM-based analyses on biomechanical exposures showed associations with CTS surgery for specific job features that have previously been implicated in the risk of CTS, such as forceful gripping, repetitive wrist movements and HAV exposure.^110^,^12^ These factors can all increase the pressure within the carpal tunnel and cause indirect ischaemic nerve changes as well as direct mechanical injury through either the effects of vibration or contact stresses.^24^ The effect of pinch grip in the development of CTS has not been fully elucidated.^19^ Of the few previous studies that have included pinch grip as a variable, results have been conflicting with some studies reporting no significant effect,^25^,^27^ while others have found an increased probability of CTS with precision-type hand grip compared with power grip^28^ or pinch grip use only among women.^29^ In our study, both pinch grip and power grip use increased the risk of subsequent CTS surgery. Our findings align with physiological studies that indicate that not only power grip but also pinch grip increases the pressure within the carpal tunnel, especially when occurring simultaneously with wrist extension.^30^

Consistent scientific evidence is also lacking regarding the independent effects of HAV exposure on CTS. ^19^ Working with vibrating tools usually occurs simultaneously with gripping the tool, and thus it is very hard to separate the effects of HAV exposure from those of hand force exertion.^14^ Interestingly, in our study, workers categorised as having no exposure to HAV still had an increased RR of CTS surgery compared with the reference group, and the risk increased only slightly among those who were exposed to HAV, and no clear distinction was seen between the low and high exposure categories. This suggests that any added effect of HAV exposure among subjects with other biomechanical exposures was minor. However, this topic requires future research using more precise exposure assessment.

Comparisons with findings from previous studies are slightly hampered by the fact that most have defined the CTS outcome as a clinical entity, sometimes corroborated by nerve conduction studies, but not necessarily involving surgical treatment. Another limitation is the fact that most previous studies have calculated ORs while our study reports RR, and these ratios diverge with increasing event rates. In our study, the RRs for the six force and posture biomechanical exposures ranged from 2.0 to 2.6 for the highest exposure categories, which we consider to be clinically significant increased risks. Our findings are in line with a previous meta-analysis on occupational risk factors for CTS, including 37 studies reporting an approximate doubling of CTS risk for the exposure variables hand force, repetition, use of vibrating tools and wrist posture.^11^ The highest exposure category for HAV in our study showed an RR of 2.3, which can be compared with a meta-analysis that described that working with vibrating tools increases the risk of CTS with an aggregated OR of 2.9 (95% CI 1.7 to 5.0).^13^ In the Danish study mentioned previously, the excess fraction of CTS due to occupational risk factors was 49% among men.^22^ Using a JEM to assign exposure, they found increased probability of CTS for subjects with high exposure to hand-related force (OR 3.8; 95% CI 3.2 to 4.3), repetitive hand movements (OR 3.2; 95% CI 2.8 to 3.8), non-neutral hand postures (OR 3.7; 95% CI 2.3 to 5.9) and HAV exposure (OR 2.4; 95% CI 2.1 to 2.8).^22^ These findings are well in line with those of our study and emphasise the potential for the prevention of CTS in the occupational setting.

Among personal factors, we found a higher risk of CTS surgery in older workers, which could be explained by cumulative occupational exposure effects, but also by intrinsic changes in nerve function with ageing. Ever-smokers had a higher RR for CTS surgery in our study; the previous literature on tobacco use is inconsistent, where some authors have described a positive association that was not found in other studies.^4 5^ We found increased risk among those with high BMI, which has also been reported previously.^5^ Separately, there was almost a linear increase in the risk of CTS surgery with decreasing height. Previous studies have also indicated increased risk with certain anthropometric variables such as short stature, short hands, wide palms and wrists with low depth in relation to width.^31^,^33^ One study focusing on genetic markers for CTS indicated a causal link between shorter height and higher risk.^6^ Thus, height could be examined more closely in the future as a proxy measure for the other hand indices and possibly serve as a constitutional predictor of CTS risk.

### Methodological concerns

The case definition used in our study could be considered both a weakness and a strength. We used a combination of the ICD-10 code for CTS and the surgical code for decompression of the peripheral median nerve, to limit the risk of including more proximal lesions than CTS, such as median nerve entrapment at the elbow or proximal forearm (ie, pronator syndrome or anterior interosseous syndrome).^34^ In addition to the surgical cases in our study, there were likely more subjects within the cohort who experienced symptoms of CTS or were clinically diagnosed but only received conservative care. Classifying these subjects as non-cases would have underestimated the impact of occupational factors on the occurrence of CTS. Instead, using the strict case definition that required a surgical procedure meant that each case had been carefully investigated and should ensure that the diagnosis was well-founded. This is important since symptom-based case definitions are less stringent and often unspecific. In fact, a previous Swedish prevalence study showed that while more than 14% of the general population reported CTS symptoms in a mail survey, less than 4% were diagnosed clinically as having CTS.^35^

Another important consideration is our use of a JEM, which is often considered the best available method for retrospective exposure assessment in cohort studies, especially when many different jobs are studied.^36^ The JEM was based on estimations of biomechanical exposures performed by occupational health and safety experts rather than being self-reported by the workers. Also, exposure assessments were performed during the health surveillance programme,^21^ and this was long before the outcomes were determined. However, the exposure assessments were based on occupational title, and intraindividual variation could not be evaluated. Also, the limited number of examinations in the health surveillance programme and the long period elapsed from the examinations until the follow-up period mandated the assumption that workers in the cohort stayed within the same occupational group from the examination at which the job title was recorded until the time of surgery, the end of the observation period or censoring. Those who changed jobs during this time frame could therefore have had completely different biomechanical exposures in their early years of employment than those assigned at their last examination. However, we know from previous studies that Swedish construction workers have often stayed within the same occupational trade group.^37^ We therefore believe that changing between trades should not have affected our results. Another concern is that some subjects could have transitioned from manual construction work to being foremen, and thus previously exposed workers would have been included in the presumed unexposed reference group. Any such errors would have led to an underestimation of RR for manual workers compared with a completely unexposed reference group. However, a sensitivity analysis using only white-collar workers and not foremen as the reference group produced similar results (online supplemental tables 2 and 3). We believe that cumulative exposure rather than recent or maximum exposure is the most appropriate measure in relation to the studied outcome. In a previous paper on the same study cohort, restricting analyses to only include exposure during the last 5 years preceding the observation period did not produce higher risk estimates for ulnar nerve entrapment.^21^ Finally, the JEM did not account for within-job changes in working conditions that could have occurred during the study period. It is possible that tasks and specific exposures might have changed from the 1970s to the 1990s, but this would not be reflected in our assessments of occupational exposure. For instance, the vibration intensity of many handheld tools has decreased as well as manual handling of building material. However, we argue that all these potential sources of exposure misclassification would be non-differential and therefore would not exaggerate the RR estimates.

There are also inherent strengths to our study. To our knowledge, it is one of the largest to date on occupational risk factors for CTS surgery. The study sample was homogenous concerning ethnicity, educational level, income and social support. We also believe that the sample was representative of the construction sector in Sweden as a whole, since the health examinations included more than 80% of the eligible construction workers. The study had a prospective design, which means that the time relation between exposure and outcome could be ascertained. We used an internal reference group within the cohort, which may have limited the available exposure contrast (as noted above) but likely also reduced confounding by socioeconomic or cultural factors. We also adjusted the analyses for personal factors and cohort effects (ie, calendar period). We believe it unlikely that any other factor (eg, socioeconomic status, psychosocial factors or work pace) would have systematically influenced the results.

### Implications

Our study suggests several potential occupational risk factors for CTS requiring surgery. Clarifying the relationship between occupational biomechanical exposures and CTS can provide employers and occupational health services with better means to prevent the occurrence of this common and disabling condition. Moreover, the fact that self-reported pain or discomfort in the hand predicted future CTS surgery could possibly be used in a preventive strategy, where recurrent screening of hand symptoms could be considered a means to detect early signs of CTS. Finally, increased knowledge about occupational risk factors can contribute to a fairer assessment regarding workers’ compensation claims.

## Conclusions

Occupational upper extremity load and postural exposures were associated with increased risk for surgical treatment for CTS in this large construction worker cohort. Preventive actions and consideration of occupation on assessment are warranted.

## Supplementary material

10.1136/oemed-2024-110008 online supplemental file 1

## Data Availability

Data are available upon reasonable request.
