# Supplementary material for: Occupational biomechanical risk factors for carpal tunnel syndrome surgery: a prospective cohort study on 203 866 Swedish male construction workers followed for 19 years
Source: Occup Environ Med. 2025 Aug 7;82(6):e110008. doi: 10.1136/oemed-2024-110008 (PMC12421095; doi:10.1136/oemed-2024-110008)
Supplement: online supplemental file 1 [file oemed-82-6-s001.docx]

**ONLINE SUPPLEMENTARY MATERIAL**

**Title:** Occupational biomechanical risk factors for carpal tunnel syndrome surgery – a prospective cohort study on 203,866 Swedish male construction workers followed for 19 years

**Authors:** Albin Stjernbrandt, Per Liv, Jennie A. Jackson, Hans Pettersson, Charlotte Lewis, Laura Punnett, and Jens Wahlström.

**Supplemental table 1.** JEM mapping for all occupational groups.

| **Occupational group** | **Intensity of upper extremity load ^a^** | **Frequency of repetitive wrist flexion and extension ^a^** | **Frequency of full wrist extension ^a^** | **Intensity of power grip ^a^** | **Frequency of pinch grip use ^a^** | **Frequency of hand-held tool use ^a^** | **Magnitude of hand-arm vibration exposure ^b^** |
| --- | --- | --- | --- | --- | --- | --- | --- |
| Asphalt workers | 2 | 1 | 1 | 1 | 1 | 1 | 1 |
| Rock workers | 3 | 1 | 1 | 3 | 1 | 3 | 3 |
| Concrete workers | 3 | 1 | 1 | 3 | 2 | 3 | 3 |
| Woodworkers | 3 | 3 | 2 | 3 | 2 | 3 | 2 |
| Bricklayers | 2 | 3 | 2 | 2 | 3 | 3 | 1 |
| Floor layers | 2 | 1 | 1 | 2 | 2 | 2 | 2 |
| Heavy machinery operators | 1 | 1 | 2 | 1 | 1 | 1 | 1 |
| Crane operators | 1 | 3 | 2 | 1 | 2 | 1 | 1 |
| Drivers | 1 | 1 | 2 | 1 | 1 | 1 | 1 |
| Glass workers | 3 | 1 | 1 | 3 | 3 | 1 | 1 |
| Insulators | 2 | 1 | 1 | 2 | 1 | 2 | 2 |
| Refrigerator technicians | 2 | 1 | 1 | 2 | 1 | 2 | 2 |
| Plumbers | 2 | 2 | 1 | 3 | 2 | 3 | 1 |
| Painters | 2 | 3 | 1 | 2 | 3 | 3 | 1 |
| Sheet metal workers | 3 | 1 | 1 | 3 | 3 | 3 | 2 |
| Electricians | 2 | 2 | 2 | 2 | 3 | 3 | 2 |
| Foremen | 1 | 1 | 1 | 1 | 1 | 1 | 1 |
| White collar workers | 1 | 1 | 1 | 1 | 1 | 1 | 1 |
| Repairers | 2 | 1 | 1 | 2 | 2 | 2 | 3 |
| Preparatory workers | 2 | 1 | 1 | 2 | 2 | 2 | 1 |
| Roofers | 2 | 1 | 1 | 2 | 2 | 3 | 1 |

^a^ 1=low, 2=moderate, 3=high.

^b^ 1=none, 2=low, 3=high.

**Supplemental table 2.** The relative risk of carpal tunnel syndrome surgery by occupational group, sorted in order of descending relative risks, using only white-collar workers (and not foremen) as reference group.

| **Occupational group ^a^** | **Total number of workers** | **Person-years** | **Number of cases** | **Incidence rate ^b^** | **Relative risk ^c^** | **95% confidence interval** |
| --- | --- | --- | --- | --- | --- | --- |
| White-collar workers | 6,499 | 69,720 | 42 | 60.2 | Reference | - |
| Wood workers | 47,416 | 691,067 | 1,183 | 171.2 | 2.9 | 2.2–4.0 |
| Floor layers | 4,122 | 59,080 | 103 | 174.3 | 2.9 | 2.0–4.2 |
| Sheet metal workers | 9,457 | 139,069 | 233 | 167.5 | 2.8 | 2.0–3.9 |
| Roofers | 1,008 | 13,284 | 23 | 173.1 | 2.7 | 1.6–4.5 |
| Bricklayers | 5,839 | 76,619 | 124 | 161.8 | 2.7 | 1.9–3.8 |
| Asphalt workers | 2,751 | 34,919 | 63 | 180.4 | 2.7 | 1.8–4.0 |
| Concrete workers | 17,905 | 232,284 | 364 | 156.7 | 2.5 | 1.8–3.4 |
| Painters | 16,856 | 241,093 | 328 | 136.1 | 2.4 | 1.7–3.3 |
| Repairers | 1,782 | 22,627 | 35 | 154.7 | 2.4 | 1.5–3.7 |
| Plumbers | 16,988 | 233,195 | 329 | 141.1 | 2.3 | 1.7–3.2 |
| Rock workers | 1,527 | 16,361 | 25 | 152.8 | 2.2 | 1.4–3.7 |
| Crane operators | 1,972 | 18,818 | 29 | 154.1 | 2.2 | 1.4–3.6 |
| Glass workers | 2,096 | 29,796 | 40 | 134.3 | 2.2 | 1.4–3.4 |
| Preparatory workers | 7,178 | 98,333 | 138 | 140.3 | 2.2 | 1.5–3.1 |
| Heavy machinery operators | 7,394 | 88,095 | 121 | 137.4 | 1.9 | 1.4–2.8 |
| Insulators | 2,033 | 29,306 | 34 | 116.0 | 1.9 | 1.4–2.8 |
| Electricians | 29,335 | 449,042 | 453 | 100.9 | 1.8 | 1.3–2.4 |
| Refrigerator technicians | 1,044 | 15,595 | 16 | 102.6 | 1.7 | 0.95–3.0 |
| Drivers | 2,637 | 31,610 | 32 | 101.2 | 1.5 | 0.9–2.3 |
| Foremen | 18,027 | 208,512 | 136 | 65.2 | 1.1 | 0.8–1.5 |

^a^ Determined by the most recent recorded data from the national occupational health surveillance program.

^b^ Per 100,000 person-years.

^c^ Adjusted for age, height, body mass index, smoking status, and time of surgery (first or second half of the observation period).

**Supplemental table 3.** The relative risk of carpal tunnel syndrome surgery in relation to occupational exposure variables, as determined by the job-exposure matrix, using only white-collar workers (and not foremen) as reference group.

| **Variable** | **Categories** | **Total number of workers** | **Person- years** | **Number of cases** | **Incidence rate ^a^** | **Relative risk ^b^** | **95% confidence interval** |
| --- | --- | --- | --- | --- | --- | --- | --- |
| White-collar workers | - | 6,499 | 69,720 | 42 | 60.2 | Reference | - |
|  |  |  |  |  |  |  |  |
| Intensity of upper extremity load | Low | 30,030 | 347,035 | 318 | 91.6 | 1.4 | 1.03–2.0 |
|  | Moderate | 88,936 | 1,273,091 | 1,646 | 129.3 | 2.2 | 1.6–2.9 |
|  | High | 78,401 | 1,108577 | 1,845 | 166.4 | 2.8 | 2.0–3.8 |
|  |  |  |  |  |  |  |  |
| Frequency of repetitive wrist flexion and extension | Low | 78,961 | 1,018,870 | 1,363 | 133.8 | 2.1 | 1.5–2.8 |
|  | Moderate | 46,323 | 682,237 | 782 | 114.6 | 1.9 | 1.4–2.6 |
|  | High | 72,083 | 1,027,597 | 1,664 | 161.9 | 2.7 | 2.0–3.7 |
|  |  |  |  |  |  |  |  |
| Frequency of full wrist extension | Low | 102,774 | 1,373,452 | 1,867 | 135.9 | 2.2 | 1.6–3.0 |
|  | Moderate | 94,593 | 1,355,251 | 1,942 | 143.3 | 2.4 | 1.7–3.2 |
|  | High | 0 | 0 | 0 | 0 | - | - |
|  |  |  |  |  |  |  |  |
| Intensity of power grip | Low | 32,781 | 381,953 | 381 | 99.8 | 1.6 | 1.1–2.1 |
|  | Moderate | 69,197 | 1,004,978 | 1,254 | 124.8 | 2.1 | 1.6–2.9 |
|  | High | 95,389 | 1,341,772 | 2,174 | 162.0 | 2.7 | 2.0–3.6 |
|  |  |  |  |  |  |  |  |
| Frequency of pinch grip use | Low | 35,413 | 424,397 | 427 | 100.6 | 1.6 | 1.1–2.2 |
|  | Moderate | 98,371 | 1,368,687 | 2,204 | 161.0 | 2.6 | 2.0–3.6 |
|  | High | 63,583 | 935,619 | 1,178 | 125.9 | 2.1 | 1.6–2.9 |
|  |  |  |  |  |  |  |  |
| Frequency of handheld tool use | Low | 34,877 | 411,749 | 421 | 102.3 | 1.6 | 1.2–2.2 |
|  | Moderate | 16,159 | 224,940 | 326 | 144.9 | 2.3 | 1.7–3.2 |
|  | High | 146,331 | 2,092,014 | 3,062 | 146.4 | 2.5 | 1.8–3.3 |
|  |  |  |  |  |  |  |  |
| Magnitude of hand-arm vibration exposure | Low | 82,746 | 1,074,272 | 1,363 | 126.9 | 2.0 | 1.5–2.8 |
|  | Moderate | 93,407 | 1,383,158 | 2,022 | 146.2 | 2.5 | 1.8–3.3 |
|  | High | 21,214 | 271,273 | 424 | 156.3 | 2.4 | 1.8–3.3 |

^a^ Per 100,000 person-years.

^b^ Adjusted for age, height, body mass index, smoking status, and time of surgery (first or second half of the observation period).


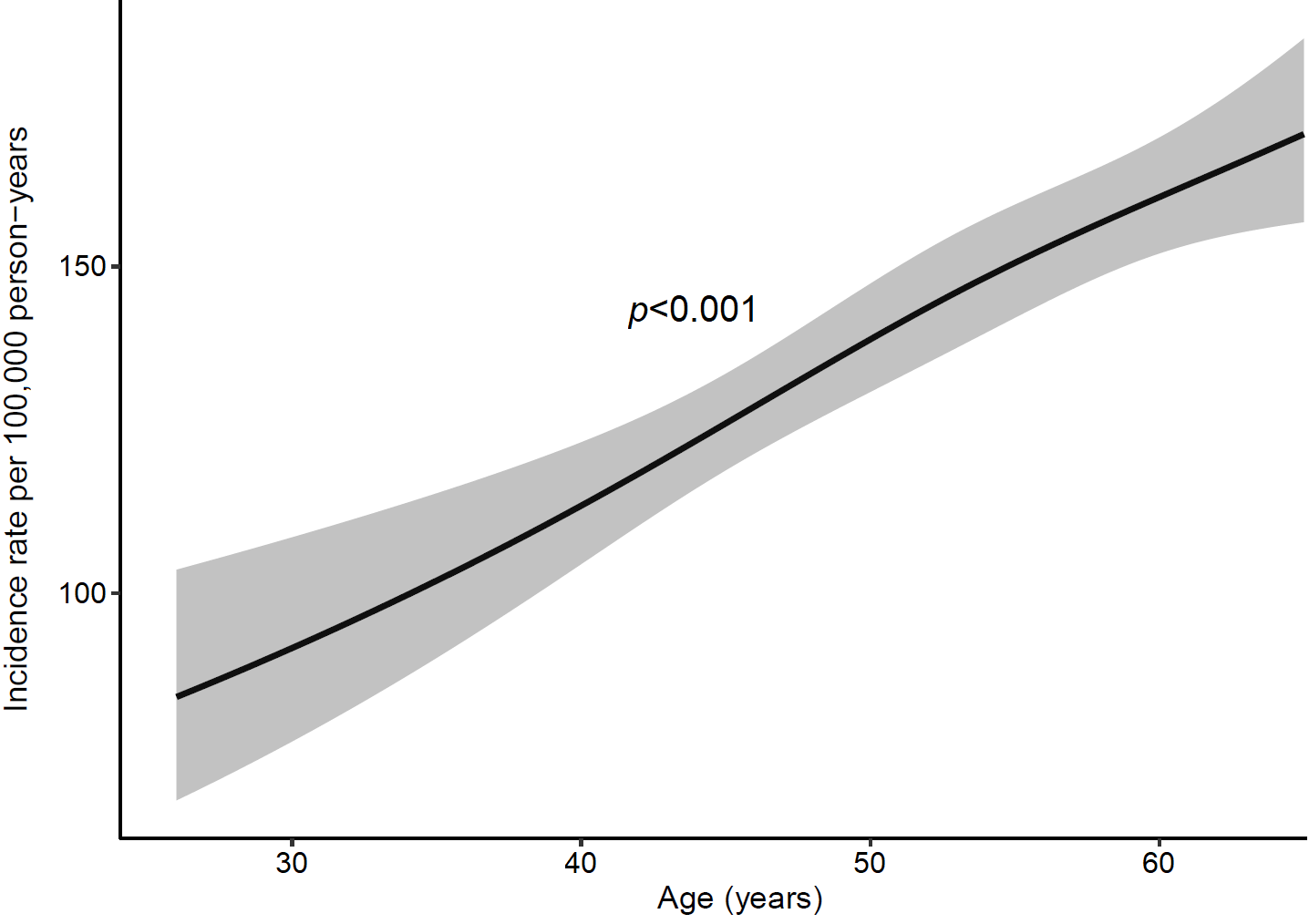


**Supplemental figure 1.** The incidence rate of surgery for carpal tunnel syndrome, modeled by age and adjusted for calendar time.
